# State-dependent behavior and alternative behavioral strategies in brown trout (*Salmo trutta* L.) fry

**DOI:** 10.1007/s00265-016-2215-y

**Published:** 2016-10-10

**Authors:** Joacim Näslund, Jörgen I. Johnsson

**Affiliations:** Department of Biological and Environmental Sciences, University of Gothenburg, Box 463, 405 30 Gothenburg, Sweden

**Keywords:** Animal personality, Behavioral syndrome, Compensatory growth, Food restriction, Mirror aggression, Open-field activity, Repeatability

## Abstract

**Abstract:**

Animals generally adjust their behavior in response to bodily state (e.g., size and energy reserves) to optimize energy intake in relation to mortality risk, weighing predation probability against the risk of starvation. Here, we investigated whether brown trout *Salmo trutta* adjust their behavior in relation to energetic status and body size during a major early-life selection bottleneck, when fast growth is important. Over two consecutive time periods (P1 and P2; 12 and 23 days, respectively), food availability was manipulated, using four different combinations of high (H) and low (L) rations (i.e., HH, HL, LH, and LL; first and second letter denoting ration during P1 and P2, respectively). Social effects were excluded through individual isolation. Following the treatment periods, fish in the HL treatment were on average 15–21 % more active than the other groups in a forced open-field test, but large within-treatment variation provided only weak statistical support for this effect. Furthermore, fish on L-ration during P2 tended to be more actively aggressive towards their mirror image than fish on H-ration. Body size was related to behavioral expression, with larger fish being more active and aggressive. Swimming activity and active aggression were positively correlated, forming a behavioral syndrome in the studied population. Based on these behavioral traits, we could also distinguish two behavioral clusters: one consisting of more active and aggressive individuals and the other consisting of less active and aggressive individuals. This indicates that brown trout fry adopt distinct behavioral strategies early in life.

**Significance statement:**

This paper provides information on the state-dependence of behavior in animals, in particular young brown trout. On the one hand, our data suggest a weak energetic state feedback where activity and aggression is increased as a response to short term food restriction. This suggests a limited scope for behavioral alterations in the face of starvation. On the other hand, body size is linked to higher activity and aggression, likely as a positive feedback between size and dominance.

The experiment was carried out during the main population survival bottleneck, and the results indicate that growth is important during this stage, as 1) behavioral compensation to increase growth is limited, and 2) growth likely increases the competitive ability. However, our data also suggests that the population separates into two clusters, based on combined scores of activity and aggression (which are positively linked within individuals). Thus, apart from an active and aggressive strategy, there seems to be another more passive behavioral strategy.

**Electronic supplementary material:**

The online version of this article (doi:10.1007/s00265-016-2215-y) contains supplementary material, which is available to authorized users.

## Introduction

Food restriction reduces body condition in animals, which may lead to energy depletion and death from starvation. It is likely that food restriction also alters behavior to mitigate the risk of starvation. For instance, green sea turtles *Chelonia mydas* in poor body condition select more profitable, but also riskier, foraging areas than turtles in good body condition (Heithaus et al. 2007). Similarly, Atlantic salmon *Salmo salar* juveniles subjected to restricted feeding increase their diurnal activity out of shelter compared to well-fed conspecifics, which may signify increased risk taking as diurnal activity likely increases the exposure to predators (Orpwood et al. 2006).

Food restriction commonly leads to a higher than normal foraging rate (hyperphagia) when food becomes available again, resulting in compensatory growth (Ali et al. 2003; Dmitriew 2011). The occurrence of hyperphagia and compensatory growth following starvation suggest that foraging and growth rates are generally submaximal under normal energetic conditions (Arendt 1997; Ali et al. 2003). The effects of food restriction on behavior are generally believed to be linked to the production-mortality trade-off hypothesis, where animals optimize their foraging behavior in relation to mortality risk (Gilliam and Fraser 1987; Werner and Anholt 1993; Fiksen and Jørgensen 2011). This trade-off could incorporate two main feedback systems (Luttbeg and Sih 2010; Sih et al. 2015). On the one hand, there is a negative “starvation-threshold” feedback consisting of starvation avoidance (SA) at the one end, and asset protection (AP) at the other (Sih 1980; Lima 1986; Pettersson and Brönmark 1993; Clark 1994; Heithaus et al. 2007; Luttbeg and Sih 2010). This negative feedback (SA-AP) lead to lower-asset individuals (i.e., with relatively low predicted fitness, e.g., small body size or low energy reserves) being more willing to accept risky situations as a consequence of having to increase their assets, while higher-asset individuals can afford to avoid risk at the expense of some of their assets (e.g., energy reserves). On the other hand, there is a positive feedback based on state-dependent safety (SDS) (Clark 1994; Luttbeg and Sih 2010). In this case, the high asset values (e.g., large energy reserves or body size) lead to higher competitive ability, and reduce risks due to predator gape-limits or increased potential swimming speed (Mittelbach 1981; Peterson and Wroblewski 1984; Werner and Gilliam 1984; Travis et al. 1985; but see Lima 1986). The influence of these feedback systems could differ in strength in different environmental contexts, e.g., depending on population density, predator abundance, predator guild composition, or ontogenetic time constraints (Ludwig and Rowe 1990). SDS and SA-AP may be elicited together, e.g., with lager individuals being more safe than smaller (SDS), but with SA-AP acting within each size class. If SA-AP is strong, then studies on individual behavioral consistency (a component of animal personality; see e.g., Bell 2007) need to take bodily state into account. Failing to do so when state does affect the behavioral consistency may lead to either a false conclusion of no consistency (when individuals’ state changes a lot between trials), or a false conclusion of consistency (when individuals’ state is consistently different due to, e.g., environmental factors unrelated to personality). In this paper, we investigate the relationships between bodily state (energy state as manipulated by recent feeding history, as well as body size) and behavior in young juvenile brown trout *Salmo trutta*. We also investigated whether behavioral variation among individuals was consistent, forming behavioral syndromes.

Our *primary aim* was to investigate state-dependent behavior in young individuals. Like in many other animals with high fecundity, the early juvenile stage of brown trout is a major selective bottleneck where individuals need to grow rapidly regardless of bodily state, due to selection against small-sized individuals through predation or competition (Elliott 1990; Degerman et al. 2001; Perez and Munch 2010). To explore whether or not these fish adjust their growth and behavior in relation to their bodily state, we manipulated food rations of individual trout and subsequently scored their behavior in standardized laboratory tests. Specifically, we tested effects of food ration on swimming activity, neophobia, and aggression. Activity and neophobia were assumed to be related to risk taking, and aggression have been found to be important to obtain a territory, which is beneficial for foraging efficiency (Elliott 1990; Johnsson and Björnsson 1994; Johnsson et al. 1999). In line with studies on older stages of salmonid fish (e.g., Johnsson et al. 1996; Nicieza and Metcalfe 1997; Höjesjö et al. 1999; Vehanen 2003; Orpwood et al. 2006), activity, neophobia, and aggression were predicted to be relatively higher in low-asset fish (i.e., fish being starved), as foraging would be important to regain lost body growth. Particularly, we predicted that the group being initially food restricted and subsequently re-fed (LH) would have the highest activity, neophobia, and aggression, as these fish were assumed to be in a compensatory growth phase. Other treatments were essentially included as controls: continuously fed (HH), continuously restricted (LL), and initially well fed followed by food restriction (HL); the HL treatment controlled food ration change (Kotrschal and Taborsky 2010). However, the above-stated general predictions regarding low-asset individuals apply to LL and HL, in relation to HH. Compensatory growth, predicted for LH, has been observed repeatedly in older juveniles of brown trout from the same population as used in this study (Johnsson and Bohlin 2006; Sundström et al. 2013; Näslund et al. 2015). Alternatively, SDS resulting from increased size may result in a general tendency for trout fry to maximize activity, neophobia, and aggression regardless of energetic state. Indeed, some studies indicate that young fish are maximizing growth with little capability to further increase their foraging efforts (Pedersen 1997; Conceição et al. 1998; Peck et al. 2014). In contrast to many previous studies, we aimed to standardize acute hunger levels, to measure effects of energetic state only.

Our *second aim* was to investigate whether brown trout fry show consistent individual differences in behavior (in the short term, over 2 days), whether different behavioral traits were correlated in the study population (indicative of behavioral syndromes, see Sih et al. 2004), and whether these traits were related to bodily state at the end of the study (energetic state or body size). Distinct personalities are often assumed to be the behavioral expressions of general life-history strategies caused by underlying differences in physiology (Koolhaas et al. 1999; Korte et al. 2005; Stamps 2007; Réale et al. 2010). The prediction was that behaviors would be correlated and repeatable, in line with previous studies of yearling brown trout (Höjesjö et al. 2011; Hoogenboom et al. 2012; Adriaenssens and Johnsson 2013; Kortet et al. 2014). However, an alternative prediction is that behavioral traits may not be correlated in a syndrome, as a previous study have suggested that behavioral syndromes may arise after the initial critical fry period, as a result of early selection for individuals fitting into the syndrome (Adriaenssens and Johnsson 2013).

Our *third aim* was to investigate whether the behavioral syndrome of brown trout fry is formed by a single continuum or several distinct clusters of consistent behavioral expression. Previous studies suggest that there are two, more or less distinct, behavioral strategies adopted by emerging salmonid fry, which differ in several traits such as activity and dispersal tendency (Héland 1999; Skoglund and Barlaup 2006). One strategy is to quickly establish and actively defend a territory (active and aggressive strategy), while the other is to hide and nocturnally disperse downstream from the nest and away from the main area of competition (passive and shy strategy). These strategies are suggestively independent of social environment, since the passive strategy is observed also in isolated fish, i.e., in absence of a social hierarchy (Héland 1999). In general discussions of animal personality, the behavioral traits are often dichotomized, characterizing individuals as belonging to one or the other end of continuous behavioral axes (e.g., fast vs. slow pace-of-life [Réale et al. 2010]; proactive vs. reactive coping style [Koolhaas et al. 1999]; “Hawk” vs. “Dove” [Korte et al. 2005]). However, the distributions of behavioral traits have rarely been explicitly investigated in empirical studies. Knowledge about trait distribution in a population is important information for future studies investigating, e.g., selection pressures on brown trout behavior (disruptive or stabilizing), and could be used in ecologically realistic individual-based models of brown trout population dynamics (Grimm and Railsback 2005).

## Materials and methods

### Study population characteristics

We used fish from a natural population of sea trout, the anadromous form of the brown trout, from the coastal stream Norumsån in Sweden (N58° 2.589′, E11° 50.759′). The adult sea trout spawns in rivers in late autumn, the eggs hatch early in the following spring, and fry emerges from the gravel in late spring (May–June) (Degerman et al. 2001). At this point, the fry start to feed and establish territories (Elliott 1994; Héland 1999). In Norumsån, juveniles normally stay in the stream for one or two summers before migrating to the sea in the following spring, typically at a size of 70–160 mm (Bohlin et al. 1993, 1996). However, depending on body condition in the previous year, up to half of the 1-year-old males, and a lower proportion of females, stay in the stream as resident adults (Dellefors and Faremo 1988; Bohlin et al. 1994, Pettersson 2002). Thus, restricted growth at early stages may have extensive effects on life-history decisions.

### Capture and housing

We captured 144 recently emerged fry on one of the stream’s main spawning grounds on June 5, 2012, using electrofishing (L-600; Lug AB, Sweden; straight DC, 200–300 V) and brought them to the laboratory. All fish were initially put in one 70-l holding aquarium, equipped with sand and plastic fanwort plants, for 7 days. During this time, we supplied the fish with pre-frozen chironomid larvae, approximately 5–10 larvae per fish and day. During the treatment period (see below), fish were housed individually in ten 55 l polypropylene storage boxes (Nordiska Plast, Sweden), each modified to contain 12 equally sized compartments (bottom area, 100 × 150 mm; water depth, 100 mm; see drawing in Electronic supplementary material, Fig. S1). Water continuously flowed through all compartments, supplied by the in-house semi-recirculating system (average temperature, 11.5 °C; range, 10.3–11.9 °C). All compartments had 5 mm of sand as bottom substrate. The boxes were covered with lids to prevent escape by jumping. Light was supplied by fluorescent tubes above the rearing boxes, with the armature being covered by black garbage bags to reduce light intensity (illuminance inside the rearing compartments was ca. 100 lx).

### Food manipulation (treatment)

At the start of the experiment, the fish were randomly split into two feeding groups (*n* = 60): high food ration (H) and restricted food ration (L); see Table 1. These rations were given over a period of 12 days (period 1, henceforth referred to as P1). At the end of P1, 12 fish had died (H, 4; L, 8). Furthermore, eight fish which had been on high ration but lost in mass were removed from the experiment as they did not fulfill the criteria for the treatment (i.e., being well fed). The two feeding groups were split in half by random assignment of the remaining fish, creating two sub-groups from each initial feeding group. One sub-group from each initial feeding group was given high food rations, and the other sub-groups were given restricted rations, see Table 1. These latter rations were provided over 23 days (period 2, henceforth referred to as P2). During P2, 11 individuals died. The food ration schedule resulted in four treatment groups (*n* denote final sample size): (1) continuous high food ration (HH; *n* = 23); (2) continuous restricted food ration (LL; *n* = 21); (3) initially high food ration, switched to restricted food ration (HL; *n* = 23); and (4) initially restricted food ration, switched to high food ration (HL; *n* = 22). The supplied food consisted of thawed chironomid larvae (Akvarieteknik, Sweden). Chironomids constitute a major part of the natural food eaten by brown trout at the early fry stage (Nilsson 1956; Skoglund and Barlaup 2006). The number of chironomids given each day was always the same for all individuals within a treatment. Thus, the smallest fish received slightly more food relative to their mass than the larger fish, but the maintenance rations should regardless represent a very restricted food intake for all fish. Food rations were based on a previous experiment (Näslund et al. 2016), and during the course of the experiment, the treatment rations were adjusted for growth and bodily condition of the treatment groups, based on daily visual inspection (Table 1). Leftover chironomids were removed using a disposable pipette the day after each feeding before the provision of new food; the pipette was also dipped in compartments without leftovers to standardize disturbance. By design, the same numbers of fish from each treatment were initially present in each rearing box.

**Table 1:** Food rations for the treatment groups during the experiment

Day of experiment		Number of chironomids per fish per day			
		HH	HL	LH	LL
0		5	5	5	5
1–12	*P1*	10	10	2	2
13–17	*P2*	10	2	10	2
18–27	*P2*	12	3	15	3
28–35	*P2*	12	4	18	4
36^a^		Satiation	Satiation	Satiation	Satiation
37^a^	Trial 1	12	4	18	4
38^a^		Satiation	Satiation	Satiation	Satiation
39^a^	Trial 2	–	–	–	–
Total during treatment (1–35)		386	192	368	96
% of HH ration		100 %	50 %	95 %	25 %

*P1* first experimental feeding period, *P2* second experimental feeding period

^a^Behavioral trial period

### Growth monitoring

We recorded wet mass (precision, 0.01 g; Kern EW 3000-2M, Kern & Sohn GmbH, Balingen, Germany) and took digital photographs (Canon EOS 40D; lens: EF-S 17–85 IS USM [at 70 mm focal length]; Canon Inc., Japan) of all fish at three time points: (1) the day before the start of the food manipulation (day 0; June 9); (2) the day we switched the food ration for the HL and LH groups (day 12); and (3) the day prior to the last day of food manipulation (day 34). Mass was recorded before feeding, leaving fish unfed for 24 h prior to the weighing. From the digital photographs, we measured fork length (from the tip of the snout to the end of the central caudal fin ray; precision, 0.1 mm) using ImageJ 1.45 (http://imagej.nih.gov/ij/). During handling, the fish were anesthetized with 2-phenoxyethanol (0.5 ml l^−1^).

Growth rate in wet mass (*M*) was analyzed as specific growth rate (SGR_M_; % change per day):

$$ {SGR}_M=100\times \left( \ln \left({M}_{t_1}\right)- \ln \left({M}_{t_0}\right)\right)\times {\left({t}_1-{t}_0\right)}^{-1.} $$

where *t*_0_ and *t*_1_ are the initial and final time-point in days, respectively. This was deemed appropriate since fish generally grow exponentially early in life (Hopkins 1992). However, the measure was corrected for initial length in statistical analyses, as SGR_M_ in itself has been shown to be negatively associated with body size, when there are no effects of dominance hierarchy (Brown 1957; Brett 1979).

Since length growth generally increases as a linear function of time in young fish (Hopkins 1992), the growth rate in fork length (*L*) was analyzed as absolute growth rate (AGR_L_; in millimeter per day):

$$ {AGR}_L=\left({L}_{t_1}-{L}_{t_0}\right)\times {\left({t}_1-{t}_0\right)}^{-1} $$.

### Growth analyses

Abbreviations for statistical methods, dependent variables and factors are found in Table 2.

**Table 2:** Descriptions of abbreviations used to describe statistical analyses

Statistical methods		
LM	Linear model	
GLM	Generalized linear model	
GLMM	Generalized linear mixed model	
ICC	Intraclass correlation	
PCA	Principal component analysis	
Dependent variables		Notes
SGR_M_	Specific growth rate in wet mass (% per day)	*a*
AGR_L_	Absolute growth rate in fork length (mm per day)	*a*
Act1; Act2	Swimming activity score, trial 1; trial 2	*b*
Neo1; Neo2	Neophobia score, trial 1; trial 2	*b*
AAggr1; AAggr2	Active aggression score, trial 1; trial 2	*b*
PAggr1; PAggr2	Passive confrontation score, trial 1; trial 2	*b*
Independent factors		Notes
TR	Food treatment. Categorical between-subject factor (fixed; four levels)	*c*
FL_I_	Initial fork length (mm) at the onset of the feeding treatment. Continuous factor	
FL_F_	Final fork length (mm) at the time of the trials. Continuous factor	
DAY	Trial day. Categorical within-subject factor (fixed; two levels)	*d*
DATE	Date of size-measurement. Categorical within-subject factor (fixed; three levels)	*e*

*a* see Materials and methods: Growth monitoring

*b* see Materials and methods: Behavioral analyses

*c* see section “Food manipulation” in Materials and methods

*d* see section “Behavioral trials” in Materials and methods

*e* see section “Growth monitoring and analyses” in Materials and methods

**Fig. 1:**
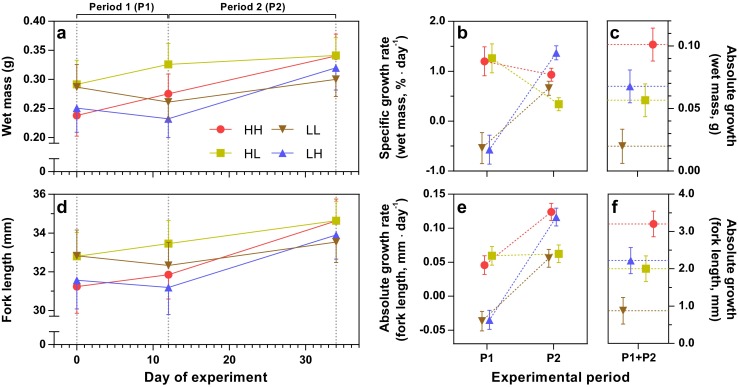
Growth patterns for the experimental fish: **a** mean wet mass; **b** specific growth rate in mass, adjusted for initial size; **c** absolute growth in mass over the experiment, adjusted for initial size; **d** mean fork length; **e** absolute growth rate in fork length, adjusted for initial size for P2); **f** absolute growth in fork length over the experiment, adjusted for initial size. *Error bars* show 95 % confidence intervals. Detailed statistics are found in the Electronic supplementary material (Section 4). For details on treatment groups (HH, HL, LH, and LL) see Table 1

Initial and final size (fork length and wet body mass) was analyzed using a GLMM (Gaussian target distribution, identity link function) with the factors TR and DATE and their interaction TR × DATE. Growth was analyzed separately for P1 and P2 using GLM (Gaussian target distribution, identity link function), including TR and *FL* at the start of each period. The interaction TR × FL was tested for significance in all growth analyses, but sequentially removed if there was low evidence for effects of this term (i.e., *p* > 0.1). Confidence intervals are presented for evaluation of treatment effects (Fig. 1); for detailed results of GLMs and GLMMs, along with contrast estimates and their *p* values, see Electronic supplementary material (Section 4, Table S2–S9).

One LL fish grew substantially faster than all other LL (SGR_M_ = 1.9 %; for comparison, see Fig. 1b) fish during P2, and was removed from all analyses investigating treatment effects, as it was likely given an erroneous ration throughout this experimental period and did not fulfill the criterion of being growth restricted.

### Behavioral trials

Behavioral trials were conducted on the second (trial 1; day 36) and fourth (trial 2; day 38) day after the end of the feeding treatment. In order to minimize the effects of hunger on our analyses, all fish were fed to satiation on the day prior to their respective trials. On trial days, fish were fed at the end of the day, with rations corresponding to the final feeding-treatment rations. On each trial day, single fish were put into opaque white trial arenas (area, 28 × 19 cm; water level, 5 cm), where behavior was recorded from above, using web-cameras (Creative VF0520; Creative Labs, Jurong East, Singapore) mounted on the ceiling. Up to nineteen fish were recorded simultaneously. Throughout the period of each trial, water temperature in the trial arena typically increased with 1.7 °C from an initial temperature of 12.0–12.3 °C.

### Trial protocol

Three consecutive behavioral tests (modified versions of the tests used in Adriaenssens and Johnsson 2013) were conducted on each trial day, with the trial order of individuals being randomized. Initially, the fish were left to swim around in the barren white environment for 15 min (*forced open-field test*). Thereafter, we lowered a novel object (trial 1: M6 hardware nut glued to a red 10 × 10-mm plastic bead; trial 2: stainless steel screw 3 × 10 mm) into one corner of the arena using a clear nylon line that was attached to the object, and subsequently left the fish for another 15 min (*novel-object test*). Finally, we introduced a mirror at one of the short sides of the container (hiding the novel object behind the mirror) and let the fish interact with its mirror image for 10 min (*mirror-aggression test*), after which the trial ended and the fish was placed back into its home tank.

### Behavioral scoring

Behavior was scored manually from recorded videos using Adobe Premier CS3 (Adobe Systems Inc., San Jose, CA, USA), with the experimenter being blind to the treatment. Abbreviations for statistical models, dependent variables, and independent factors are found in Table 2.

**Fig. 2:**
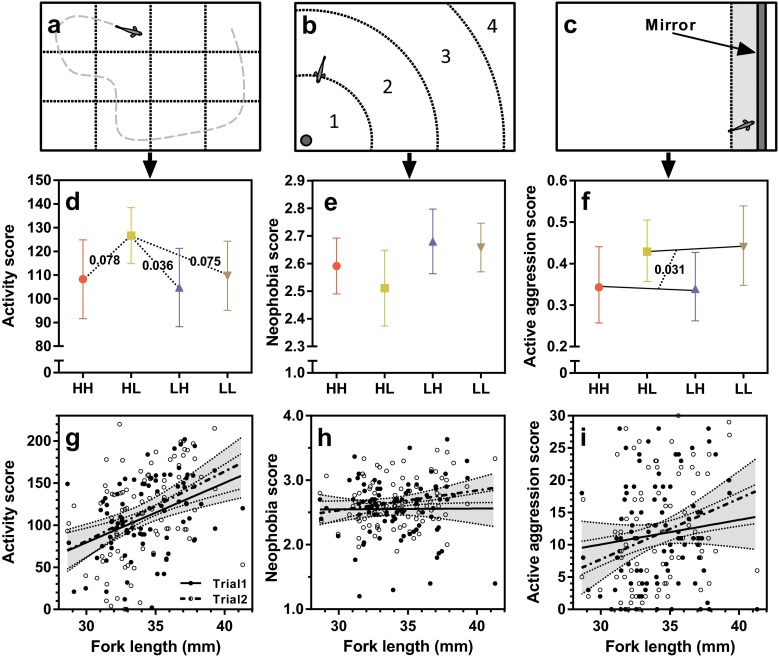
Results from the behavioral trials. *First panel*—row **a**–**c**: top-view schematic illustrations of the behavioral arenas for **a** forced open-field test, **b** novel-object test (*numbers* indicate distance-zones, as described in Materials and Methods), and **c** mirror-aggression test (*dark gray zone*: mirror; *light gray zone*: “confrontation zone”). Definitions of aggression based on fish position relative to the mirror within the confrontation zone are graphically presented in the Electronic supplementary material, Fig. S2. *Second panel*—row **d**–**f**: estimated means, with 95 % confidence intervals, based on the GLMMs (i.e., combining both behavioral trials) for **d** activity score (significant and trend contrasts connected with *dotted lines* and *p* values), **e** neophobia score, and **f** active aggression score (*dotted line* indicates significant difference in ad hoc analysis combining HH and LH, and LH and LL, along with *p* value). *Third panel*—row **e**–**i**: body size effects on **g** activity score, **h** neophobia score, and **i** active aggression score. *Gray areas* show 95 % confidence limits. For details on treatment groups (HH, HL, LH, and LL), see Table 1. The *fish symbol* represents the approximate size of a subject fish in relation to the arena

In the *forced open-field test*, we scored swimming activity (*Act1* and *Act2*; Table 2). The trial arena was divided into a grid of 12 equal-sized rectangles (70 × 63.3 mm; Fig. 2a). The number of times the whole body of the fish crossed the lines, between the 10th and 15th minute after the release into the arena, was recorded as a measure of activity. The initial 10 min were discarded as most fry tend to freeze for some time when placed into a novel environment (the vast majority freeze for <10 min).

In the *novel-object test*, we scored neophilia as a measure of boldness-like behavior (*Neo1*and *Neo2*; Table 2). Based on the distance from the novel object, four zones were defined (Fig. 2b): zone 1 (0–84-mm distance), zone 2 (85–169-mm distance), zone 3 (170–254-mm distance), and zone 4 (>255-mm distance). The zone number in which the eyes of the fish was located was scored every tenth second between the 10th and 15th minute following the introduction of the novel object (the first 10 min were discarded, as many fish tend to freeze when inserting the novel object). The average score was used as a measure of neophobia.

In the *mirror-aggression test*, we scored aggression towards the mirror image. A “confrontation zone” was defined as the area within 3-cm distance from the mirror (Fig. 2c). If the fish was inside this zone with its head, and its body was not facing away from the mirror at an angle of >45°, it was scored as a confrontation. If the fish was swimming actively against the mirror, or swimming towards the mirror at an angle of >45° inside the confrontation zone, the behavior was classified as active aggression (*AAggr1* and *AAggr2*; Table 2). If the fish was inside the zone but not moving, or it faced the mirror at an angle ≤45° or ≤45° away from the mirror, the behavior was classified as passive confrontation (*PAggr1* and *PAggr2*; Table 2). The position of each fish was scored every tenth second between the 5th and the 10th minute after the mirror was inserted into the arena, and the total number of active or passive scores were summed up and used in analyses; higher values indicating more occurrences of a given class of aggressive behavior. For a graphical illustration of the definitions of *AAggr* and *PAggr*, see Electronic supplementary material (Section 2, Fig. S2).

Given that the acute hormonal stress response in salmonids commonly lasts for at least 2 h following handling (Pickering et al. 1982), the fish should be considered being tested in a stressed state.

In all cases, lines and zones used to score behavior in the trial arenas were drawn on plastic film which was put on the computer LCD-monitor when analyzing the recorded films.

### Behavioral analyses

Statistical analyses were conducted in SPSS 22 (IMB Corp., USA), if not stated otherwise. Behavioral scores from each test were analyzed using GLMMs (covariance type: compound symmetry; robust covariance estimates; residual method for degrees of freedom estimation). The *Act*-GLMM was based on Gaussian target distribution and identity link function, while *Neo*- and *AAggr*-GLMMs were based on binomial target distribution and logit link function. Factors included in the models were TR, DAY, and FL_F_, as well as fish identity as a random factor. Initially, we also included the interactions TR × DAY and TR × FL_F_, but these interactions were not significant in any of the analyses (all *p* > 0.2) and therefore removed from the final models. Pairwise contrasts for fixed factors were checked if *p* ≤ 0.1. From the results of the *AAggr*-GLMM*,* a pattern occurred where fish ending on low ration seemed to be more aggressive. As an ad hoc analysis, we pooled the TR-levels HH and LH, and HL and LL, and ran the model again. In addition, as there was substantial variation in growth rate within treatment groups, we conducted complimentary analyses where we modeled behavioral scores as linear functions of specific growth rate during P2, without including treatment group as a factor (presented in Electronic supplementary material, Section 5). For GLMMs of *Act* and *AAggr* the final model was also run using FL_I_ instead of FL_F_, to explore the effect of initial size. *Neo-*scores were not further analyzed as the novel-object trial did not appear to result in any informative behaviors with respect to neophobia (see Electronic supplementary material, Section 3). The FL_I_ and FL_F_ models were compared using the difference in Akaike Information Criterion adjusted for small sample sizes (∆AIC_C_; smaller AIC_C_ is a model with better fit).

Repeatability of the scored behaviors was analyzed by ICC, using the “psych” package (Revelle 2015) in R 3.0.3 (R Core Team 2014).

The behavioral scores (Table 3) were combined into principal components in a PCA, using the correlation matrix. *Neo1* and *Neo2* were not included in the PCA (see Electronic supplementary material, Section 3). It can be noted that if included, these variables would load in a separate component, *Neo1* positively and *Neo2* negatively (data not shown). Out of the confrontation scores, we chose to include only *AAggr1* and *AAggr2* in the PCA (for details see Results: Aggression). The component obtained from the PCA, including *Act1*, *Act2*, *AAggr1*, and *AAggr2*, was analyzed using a GLM (Gaussian target distribution, identity link function), including TR and FL_F_; the interaction was initially included, but removed in the final analysis as it was non-significant (*p* = 0.3).

**Table 3:** Relationships among behavioral variables

Correlation matrix									Principal component analysis	
*N* = 90	*Act1*	*Act2*	*AAggr1*	*AAggr2*	*PAggr1*	*PAggr2*	*Neo1*	*Neo2*	Communalities	PC1 Factor loadings
*Act1*	–	***	**	NS	¤	NS	NS	¤	0.499	0.706
*Act2*	**0.439**	–	**	**	*	NS	NS	NS	0.594	0.771
*AAggr1*	**0.335**	**0.290**	–	**	***	¤	NS	NS	0.462	0.680
*AAggr2*	0.172	**0.363**	**0.300**	–	¤	***	NS	NS	0.401	0.633
*PAggr1*	−0.180	**−0.224**	**−0.507**	−0.187	–	*	NS	NS	–	–
*PAggr2*	−0.077	−0.131	−0.187	−**0.464**	**0.233**	–	NS	NS	–	–
*Neo1*	−0.043	0.173	0.038	−0.030	−0.074	0.072	–	NS	–	–
*Neo2*	0.192	−0.003	0.151	−0.003	−0.023	0.140	−0.058	–	–	–

Pearson correlation coefficient *r* (left table, below diagonal); significance *p* (left table, above diagonal); principle component analysis summary (right table)

Significant correlations are marked bold

*Act* swimming activity, *AAggr* active aggression, *PAggr* passive confrontation, *Boldn* neophobia, *1* first trial, *2* second trial

¤ = *p* < 0.1; * = *p* < 0.05; ** = *p* < 0.01; *** = *p* < 0.001; *NS* = not significant, *p* > 0.1

To investigate whether distinct behavioral groups could be discerned, we used the TwoStep cluster analysis (distance measure: log-likelihood), set to automatically categorize a number of clusters (maximally five) (SPSS Inc. 2001). The cluster analysis was based on the variables *Act1*, *Act2*, *Aggr1*, and *Aggr2*. Detected clusters were analyzed using binomial GLM (logit link function), including TR and FL_F_. Furthermore, to investigate whether detected clusters were set already prior to the experiment, we analyzed the cluster assignment using a binomial GLM with only initial body size (i.e., fork length prior to the onset of the feeding treatments) as a factor.

### Ethical note

Food rations where continuously assessed for adequacy with respect to fish survival, based on visual inspection of fish condition, behavior, and mortality. Although most fish fed on the provided food from the first day in the lab, some fish never started to feed which resulted in mortality. Such failure of feeding in some young salmonid fry is commonly noted in lab and hatchery environments (JN and JIJ personal observations).

## Results

Electronic supplementary tables and figures are referred to as Table S*X* and Fig. S*X*, respectively, where *X* refers to the number of the table or figure.

### Growth

The initial mean sizes of HL and LL groups were slightly, but significantly, larger than the HH and LH groups and as a consequence there was no significant differences among groups in size at the end of the treatment (wet mass: Fig. 1a, Table S2, S3; fork length: Fig. 1d, Table S6, S7). During P1, the growth rates were faster for fish on high ration; in general, high ration fish showed positive growth, while low ration fish showed negative growth (SGR_M_: Fig. 1b, Table S4; AGR_L_: Fig. 1e; Table S8). During P2, all treatment groups differed in SGR_M_, with the LH group growing at the fastest rate: LH > HH > LL > HL (Fig. 1b, Table S4). For AGR_L_ in P2, the high ration groups grew faster than low ration fish: HH ≈ LH > HL ≈ LL (Fig. 1e, Table S8). Looking at the absolute growth over the whole experiment (P1 + P2), HH grew most rapidly, in order followed by LH, HL, and LL (wet mass: Fig. 1c, Table S5; fork length: Fig. 1f, Table S9).

### Open-field activity

Body size had a significant effect on swimming activity, where larger fish were more active (FL_F_: *F*_1,172_ = 19.301; *p* < 0.0001; Fig. 2g). In the GLMM, treatment was not a significant factor (TR: *F*_3,172_ = 2.115; *p* = 0.099), but pairwise contrasts suggested that the HL group tended to be more active (HL vs. LH [21 % higher]: *p* = 0.036; HL vs. HH [17 % higher]: *p* = 0.079; HL vs LL [15 % higher]: *p* = 0.076) (Fig. 2d). Trial day had no significant effect (DAY: *F*_1,175_ = 1.544; *p* = 0.216). Regression analyses indicated that there were negative effects of specific growth rate on activity during P2 (Fig. S3).

Analysis of the activity GLMM based on FL_I_, instead of FL_F_ *,* resulted in a significant effect of treatment (FL_I_: *F*_1,172_ = 19.642, *p* < 0.0001; TR: *F*_3,172_ = 2.858; *p* = 0.039). Comparisons of the models gave ∆AIC_C_ = 0.94, with FL_F_ *<* FL_I_.

Swimming activity was generally repeatable (Table 4). However, repeatability seemed to be higher for HL and LH fish than for HH and LL, albeit with overlapping confidence intervals for *ICC*.

**Table 4:** Repeatability of behaviors as indicated by the intraclass correlation coefficient (ICC)

		Activity		Neophobia		Active aggression	
	*N*	ICC	*F*	ICC	*F*	ICC	*F*
Overall	90	**0.43** *** (0.25–0.58)	2.5	−0.066 (−0.27–0.14)	0.88	**0.30** ** (0.11–0.48)	1.9
HH	23	0.25 (−0.16–0.60)	1.7	−0.31 (−0.63–0.11)	0.53	**0.48** ** (0.11–0.74)	2.9
HL	23	**0.59** *** (0.25–0.80)	3.9	0.062 (−0.35–0.45)	1.1	0.048 (−0.36–0.44)	1.1
LH	22	**0.68** *** (0.38–0.85)	5.3	0.033 (−0.38–0.44)	1.1	0.23 (−0.20–0.59)	1.6
LL	21	0.22 (−0.22–0.59)	1.5	−0.078 (−0.48–0.35)	0.86	**0.47** * (0.072–0.75)	2.8

Numbers within brackets denote 95 % confidence interval of ICC. Significant ICCs are bold

For details on treatment groups (HH, HL, LH and LL) see Table 1

*N* final sample size, *F F* statistic

* = *p* ≤ 0.05; ** = *p* ≤ 0.01; *** = *p* ≤ 0.001

### Novel-object neophobia

No significant treatment effect was detected (TR: *F*_3,172_ = 1.446; *p* = 0.231) (Fig. 2e), neither was there any effect of body size (FL_F_: *F*_1,172_ = 2.236; *p* = 0.137) (Fig. 2h). Fish tended to be slightly further away from the novel object on the second trial day compared to the first trial day (DAY: *F*_1,172_ = 3.092; *p* = 0.080). Regression analyses did not indicate any effects of specific growth rate during P2 of the feeding-treatment period (*R* ^2^ ≤ 0.02, *p* > 0.18).

Individual neophobia scoring was not found to be repeatable between the two trial days (Table 4).

In general, scoring of neophobia was found to be largely reflecting a random swimming pattern for most individuals; i.e., for the majority of the individuals, the number of times a fish was found in each zone did not deviate from what was expected based on the size of each zone (for analyses and further discussion see Electronic supplementary material, Section 3).

### Mirror aggression

Total confrontation levels towards the mirror (i.e., *AAggr* + *PAggr*) were generally very high and close to the maximum score (Fig. S4), leading to the *PAggr1* and *PAggr2* being largely complementarily, negatively correlated, to *AAggr1* and *AAggr2*, respectively (this is the reason why we only included *AAggr* in the PCA and why we only report results on *AAggr* *;* for illustration of *PAggr* scores see Fig. S4). For active aggression scores, no significant effects were detected for treatment (TR: *F*_3,172_ = 1.465; *p* = 0.226) or trial day (DAY: *F*_1,172_ = 0.001; *p* = 0.974) (Fig. 2f). Larger fish were more aggressive (FL_F_: *F*_1,175_ = 5.857; *p* = 0.017) (Fig. 2i). Pooling fish with respect to the ration given during P2 (i.e., HH + LH, and HL + LL) revealed that fish reared on low ration during P2 were more aggressive, as well as the same general size effect (FL_F_: *F*_1,172_ = 5.821, *p* = 0.017; TR_Pooled_: *F*_1,174_ = 5.619, *p* = 0.019) (Fig. 2f). Regression analyses also indicated that there was a negative effect of specific growth rate on aggression during P2 (Fig. S3).

Analysis of the GLMM for active aggression based on FL_I_, instead of FL_F_ *,* resulted in no effect of treatment (FL_I_: *F*_1,174_ = 7.129, *p* = 0.008; TR: *F*_3,174_ = 0.809; *p* = 0.491). Comparisons of the models gave ∆AIC_C_ = 0.22, with FL_F_-model *<* FL_I_-model. For the data being pooled based on P2, there was no effect of treatment (FL_I_: *F*_1,174_ = 7.074, *p* = 0.009; TR_*pooled*_: *F*_3,174_ = 2.562; *p* = 0.111). Comparisons of the models gave ∆AIC_C_ = 0.44, with FL_F_ *<* FL_I_.

Active aggression was repeatable overall (Table 4). However, repeatability seemed to be higher for HH and LL fish than for HL and LH, albeit with overlapping confidence intervals for ICC (Table 4).

### Principal component analysis

In the PCA we extracted the first component (PC1) for further analysis, as both Cattell’s scree test and the Kaiser–Guttman criterion (eigenvalue > 1) signaled that only one component was informative. All included variables loaded positively on PC1 (see correlation matrix, communalities and factor loadings in Table 3). Thus, higher values of swimming activity and active aggression were represented by higher values of PC1. PC1 explained 48.9 % of the variation in the included data and the eigenvalue was 1.96. Sampling adequacy as indicated by the KMO test (0.649) and Bartlett’s sphericity test (*p* < 0.001) was regarded as acceptable, but results should be treated with some caution due to the KMO value being <0.7 (following Budaev 2010).

**Fig. 3:**
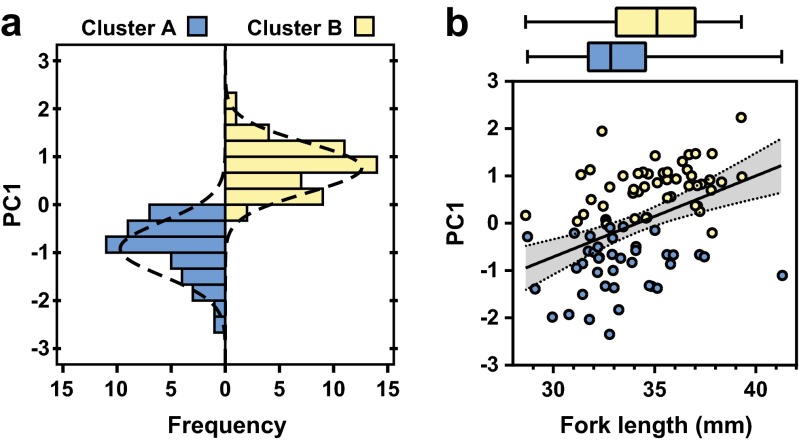
Clustering of behavioral types: **a** distribution of individuals into the two clusters in relation to their score of the extracted principal component, PC1 (*Cluster A* = less active and less aggressive; *Cluster B* = more active and more aggressive); **b** relationship between PC1 and body size (fork length). *Box-plots on top of the graph* show the fork length of the two clusters; *box hinges* show the first and third quartile, the *line inside the box* shows the second quartile (median), and the *whiskers* show minimum and maximum values. Regression line with 95 % confidence interval is shown for both clusters combined

Given the factor loadings from the PCA (Table 3), PC1 is indicating the presence of a behavioral syndrome between swimming activity and active aggression in the subject fish. The PC1 scores were not significantly different among treatments (TR: Wald *χ* ^2^ = 5.9; *df* = 3; *p* = 0.117), but higher scores were associated with longer bodies (FL_F_: Wald *χ* ^2^ = 20.235; *df* = 1; *p* < 0.001) (Fig. 3b), indicating that larger fish were more active and aggressive.

### Cluster analysis

Two behavioral groups were detected in the cluster analysis. In general, lower activity and lower aggression were associated with one cluster (cluster A, 44.9 % of individuals, Fig. 3a), and higher activity and higher aggression were associated with the other cluster (cluster B, 55.1 % of individuals, Fig. 3a). In concordance with the other results on activity and aggression, larger body size increased the probability of being assigned to cluster B (Fig. 3b) (FL_F_: Wald *χ* ^2^ = 10.685; *df* = 1; *p* = 0.001). Treatment group did not affect the probability of being assigned to a particular cluster (TR: Wald *χ* ^2^ = 3.552; *df* = 3; *p* = 0.314). Behavioral clusters appeared to be defined already prior to the onset of the experiment, as FL_I_ alone was a significant predictor of cluster assignment (Wald *χ* ^2^ = 11.520; *df* = 1; *p* = 0.001, see Fig. S4).

## Discussion

### Effects of feeding history and body size on activity and aggression

The results presented here provide some evidence, albeit notably weak, for state-dependent behavior in brown trout fry, but not following the predicted pattern. We predicted that the LH group (initially starved and subsequently re-fed at high rations), which was assumed to have entered a compensatory growth phase, would be more active due to being in a hyperphagic state, but this effect could not be confirmed. Instead, we found that the treatment group with a negative change in food ration in P2 (HL) tended to be more active in the open-field test than the other groups. We also found that food-restricted fish in P2 (i.e., HL + LL treatments pooled) showed slightly higher average levels of active aggression than fish fed high rations. Higher aggression may, in this case, reflect higher motivation to obtain and defend potential resources as aggressive rejection of competitors will increase the per capita resource availability within the individual’s home range. This is in conflict with results in Hoogenboom et al. (2012), where no effects among trout of similar age were detected. However, the fish in their study were scored in groups which may have affected aggression levels of subordinate fish. Nicieza and Metcalfe (1997) showed that older juveniles of Atlantic salmon increased aggression after being food restricted, which is in line with our findings. The prediction that initially starved and subsequently re-fed fish should be more aggressive than all other groups was not realized. Both activity and aggression were negatively correlated with growth rate during P2, albeit with relatively low *R* ^2^ values, indicating large inter-individual variation (Fig. S3). Smaller trout in general have faster growth rate (Jonsson and Jonsson 2011), as long as they are not being suppressed by dominant individuals (e.g., Brown 1957). Here, smaller fish were indeed growing faster, as expected by the fact that the fish were reared without competition for food. The finding that larger individuals were generally more active and more aggressive indicates that larger fish are more likely to belong to a more territorial behavioral type (i.e., cluster B in this study, see further discussion below).

No effects were detected for the behavior in the novel-object test. In fact, this test seemed to be largely uninformative in the way it was carried out here (see ESM, Section 3). It should be noted here that other designs of novel-object tests for recently emerged brown trout fry have proved to be useful (e.g., Sundström et al. 2004).

Overall the effects of treatment appeared to be relatively small, compared to the general behavioral expression, in agreement with another recent study on the same life-stage of brown trout from the same population (Näslund et al. 2016). Thus our results suggest that behavioral types of brown trout fry are set very early in life, possibly through genetic or epigenetic mechanisms, and the scope for adjustments of behavior is limited in the early-life stage. Furthermore, the limited scope for increased activity and aggression suggests that fry are under general pressure to attain a larger size, to avoid predation and increase competitive ability. Similar results have been obtained for juvenile stages of other fish species (e.g., Peck et al. 2014), as well as for larval insects (Brodin and Drotz 2011). Early survival of brown trout is largely dependent on whether the fish can attain a territory or not during a critical period, which corresponds to the experimental period for this study, and is negatively influenced by increased population density (Elliott 1990). It should be noted that the fish were not stimulated by any predator models during trials, and thus the conclusion that state-dependent safety is of large importance for the trout fry behavior may be less valid when individuals perceive direct predation risk. Other studies have shown that salmonid juveniles (slightly larger than our trout, and thus with more energy reserves) rely on asset protection, i.e., larger fish take fewer risks, when directly attacked by model predators (Reinhardt and Healey 1999).

### Behavioral types in brown trout fry

The brown trout fry showed individual consistencies in swimming activity and aggression at similar levels as previously reported for this species (Hoogenboom et al. 2012; Adriaenssens and Johnsson 2013; Kortet et al. 2014; Wengström et al. 2016).

Activity and aggression were generally positively correlated in the brown trout fry, forming a behavioral syndrome which has also been observed in juveniles of European eel *Anguilla anguilla* (Geffroy et al. 2015), and in adults of several fish species (reviewed in Conrad et al. 2011). When adding the same behavioral variables into a cluster analysis, two general clusters could be discerned—one with lower activity and aggression (cluster A), and one with higher activity and aggression (cluster B). The clustering of two general behavioral types is in line with the literature describing the biology of early brown trout stages, where two behavioral groups are discerned when the fry emerges from the spawning gravel. One group takes station close to the nest, and the other, having delayed formation of static swimming behavior, drift downstream away from the nest (Cuinat and Héland 1979; Héland 1999). The downstream drifters have been suggested to constitute a group of individuals with the strategy of forming territories in areas where there is less competition (Héland 1999; Skoglund and Barlaup 2006). Trout fry show these different behaviors even if reared in isolation (Héland 1999), a finding which is supported by our results. Several studies show that the early emerging salmonid fry are the ones taking station close to the nests and become dominant over later emerging fry (Mason and Chapman 1965; Chandler and Bjornn 1988; Metcalfe and Thorpe 1992). This dominance could potentially lead to a size advantage during the rest of the juvenile stage and thereby earlier smoltification (i.e., the ontogenetic transformation for seaward migration), as shown in hatchery studies (Metcalfe and Thorpe 1992). Dominant fish can choose the best foraging grounds, and also have precedence in choosing when to forage, and can thereby optimize food intake in relation to risk (Alanärä et al. 2001). Some evidence suggests that early emergers have basal higher metabolic rate, which could lead to higher activity levels (Metcalfe et al. 1995). This, in turn, would further support the inference that the active group is constituted of early emergers. Similar strategies are also found in wild brook char *Salvelinus fontinalis* fry, but in this species, the strategies appear to be associated with stress reactivity (i.e., cortisol expression) (Farwell and McLaughlin 2009; Farwell et al. 2014).

In some cases, a passive strategy may not be viable during the early critical period, as indicated by high mortalities in non-territorial fry in their first months of life in the Black Brows Beck, Britain (Elliott 1990). In other cases, like in the tributaries to the Norwegian river Daleelva, non-territorial drifting fry do not seem to starve and may thus not be outcompeted; instead this appears to be a specific dispersal strategy (Skoglund and Barlaup 2006). The possibility of coexistence of different behavioral types is likely positively influenced by territory availability and environmental complexity (Höjesjö et al. 2004; Hoogenboom et al. 2012; Reid et al. 2012), which likely differ among study sites and over time. The different clusters of behavioral types could be a result of frequency-dependent selection based on underlying physiological mechanisms (e.g., metabolic rate or stress reactivity) as modeled by Wolf and McNamara (2012). However, studies on young hatchery reared salmonids have indicated that agonistic behavior, which is part of the behavioral syndrome in our study, show low heritability (Vøllestad and Quinn 2003; Kortet et al. 2014). Still, artificial selection programs seem to be able to create genetic strains with altered aggression levels compared to wild salmonid populations, indicating that there actually is a genetic component for the behavioral expression (Huntingford and Adams 2005). Substantial among-sibling variation in behavior has previously been found in brown trout, attributed to the location of the eggs in the egg sac and possibly pre-natal hormone exposure (Burton et al. 2011, 2013). Thus, behavioral strategies of individual fry may be depending on embryonal environment, which can vary within females (Jonsson and Jonsson 2014). For instance, within-female egg size variation in southern pygmy perch *Nannoperca australis* can be influenced by environmental predictability, with higher variation in unpredictable environments (Morrongiello et al. 2012). If female investment into an egg affect behavior of the hatched fry, e.g., through effects on metabolic rate (Régnier et al. 2012), then higher size variation in unpredictable environments may be an indication of bet-hedging were different behavioral types perform well in different situations, utilizing different niches, or different competitive strategies (e.g., Grant and Noakes 1987; Skoglund and Barlaup 2006; Závorka et al. 2015). In this way, the offspring from a single female may have a wider total niche breadth. Given the many non-genetic factors which can affect offspring behavior, the frequency of behavioral types in a population may be an effect of selection for intra-female variation in offspring phenotypes and fine-tuned each generation through environmental effects, rather than being an effect of direct genetic inheritance of specific behavioral traits. An alternative possibility is that the clustering depends on some natural dichotomy present in the species, such as sex. However, a recent study has shown that there, at least, are no sex differences in energetic content, metabolic rate or emergence timing from the spawning gravel in brown trout fry (Régnier et al. 2015).

Interestingly, the treatment groups tended to differ in repeatability of these traits. Regarding activity, the groups which experienced a switch in their food ration (HL and LH) showed relatively stronger repeatability than the stable ration groups (HH and LL). Repeatability in the latter two groups was not statistically significant, although showing similar patterns as the former two groups. Previous studies have shown that environmental factors can affect the strength of personality traits (e.g., behavioral syndromes being stronger in the presence of a predator; Bell and Sih 2007) and cognitive abilities (e.g., higher ability when food rations have changed during the juvenile stage; Kotrschal and Taborsky 2010). Possibly, stability of food ration may affect the consistency of behavioral traits. Further investigation into the strength of repeatability in different environments is warranted.

It is not yet known whether brown trout retain their behavioral strategy, or personality, over longer time-periods (for similar issues see, e.g., Groothuis and Trillmich 2011). Possibly, if the low-activity fish retain their passive behavior over time, their performance may rival that of more active individuals at later life-stages (see, e.g., Adriaenssens and Johnsson 2010; Závorka et al. 2015).

### Experimental caveats

Our findings have caveats which are important to recognize for the interpretation of the experimental results and to identify where future research efforts could be directed.

Firstly, the pre-trial ad libitum ration is a major shift in food availability for fish on low ration. Thus, for the HL and LL groups, this change may possibly lead to a positive contrast effect, which means that fish experiencing a positive change in food abundance may increase their foraging efforts and alter associated behaviors (McNamara et al. 2013). Consequently, the HL and LL fish may have increased their activity as a response to the change of ration the day before trials. However, it is not known how long these contrast effects last, so they may have disappeared the following day, since no more food was given before trials. Furthermore, the fish were trialed in environments (trial arenas) different from the holding tank, and there is no information about food availability associated to the trial arena itself. Thus, the fish are naïve with respect to information about the likelihood of finding food in the trial arenas. Future studies may investigate the presence and duration of contrast effects using other food ration schedules, where fish from both high and low ration treatments either switches ration, or remain on the same ration prior to treatment (see McNamara et al. 2013).

Secondly, the results may also depend on the initial skew in body size of the fish surviving until the behavioral trials (see Fig. 1a, d). This effect was unexpected as a previous study, with practically the same feeding design, did not produce this effect (Näslund et al. 2016). The HL and LL fish were initially larger, and if activity is associated with initial size, higher activity in these groups may be associated with a general size effect where larger fish are more active. The lack of initial trials makes it impossible to discuss individual changes over the experimental period. Future studies can include initial trials to look closer into individual change due to treatment. It should be noted that the size effect is part of our main results, and consequently, the conclusion that larger fish are generally more active remains.

## Conclusions

Based on our results, we argue that behavior in brown trout fry can be influenced by recent food availability, albeit with effects being relatively weak due to inter-individual variation. Size was associated with behavior, with larger fish being more active and more actively aggressive on average. We found evidence for both short-term consistent individual differences in activity and active aggression, and a behavioral syndrome where activity and active aggression were positively correlated in the subject population. Finally, two distinct behavioral groups could be discerned despite elimination of social hierarchy effects for a month prior to behavioral trials, suggesting two general behavioral strategies in brown trout fry.

## Electronic supplementary material

ESM 1 (DOCX 951 kb)
