# Supplementary material for: State-dependent behavior and alternative behavioral strategies in brown trout (Salmo trutta L.) fry
Source: Behav Ecol Sociobiol. 2016 Oct 10;70(12):2111–25. doi: 10.1007/s00265-016-2215-y (PMC5102978; doi:10.1007/s00265-016-2215-y)
Supplement: Supplementary file 1 — (DOCX 951 kb) [file 265_2016_2215_MOESM1_ESM.docx]

**Electronic supplementary material: State-dependent behavior and behavioral types in brown trout (*Salmo trutta* L.) fry**

***Behavioral Ecology and Sociobiology***

**J. Näslund^*^ and J. I. Johnsson**

Department of Biological and Environmental Sciences, University of Gothenburg, Gothenburg, Sweden

^*^ Corresponding author: joacim.naslund@bioenv.gu.se

Contents

[1. Rearing boxes 2](#_Toc459208865)

[2. Definition of aggressive postures 3](#_Toc459208866)

[3. Neophobia test: validation 4](#_Toc459208867)

[4. Size and growth: detailed statistical results 6](#_Toc459208868)

[Size: wet mass (g) (GLMM) 6](#_Toc459208869)

[Growth rate: *SGR_M_*, specific growth rate (wet mass, % per day) (GLMs) 7](#_Toc459208870)

[Total growth: absolute growth in wet mass (g) (GLM) 8](#_Toc459208871)

[Size: fork length (mm) (GLMM) 9](#_Toc459208872)

[Growth rate: *ΔL,* absolute growth rate in fork length (mm per day) (GLMs) 10](#_Toc459208873)

[Total growth: absolute growth in fork length (mm) (GLM) 11](#_Toc459208874)

[5. Complementary analyses of behavior 12](#_Toc459208875)

[Growth rate vs. behavior: analyses using growth rate as continuous factor 12](#_Toc459208876)

[Mirror confrontation scores: total confrontation, passive- and active aggression 13](#_Toc459208877)

[Cluster assignment in relation to initial and final size 13](#_Toc459208878)

# Rearing boxes

**
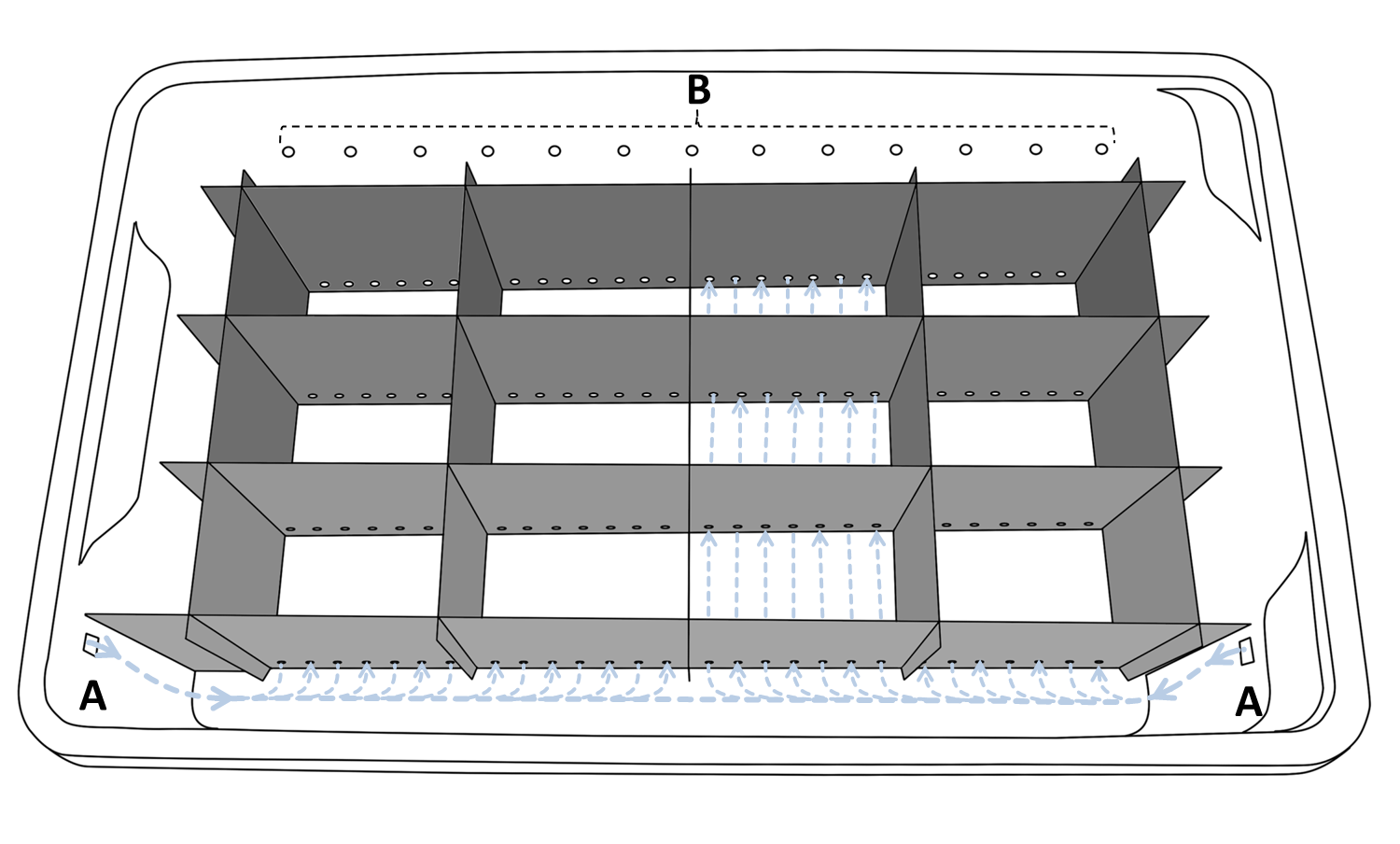
**

**Figure S1.** Illustration of a rearing box used for individual rearing of brown trout fry (as viewed from above). Blue arrows show the flow through the fish compartments, from the inlets (A) to the outlets (B). The box was covered by a lid.

# Definition of aggressive postures

**
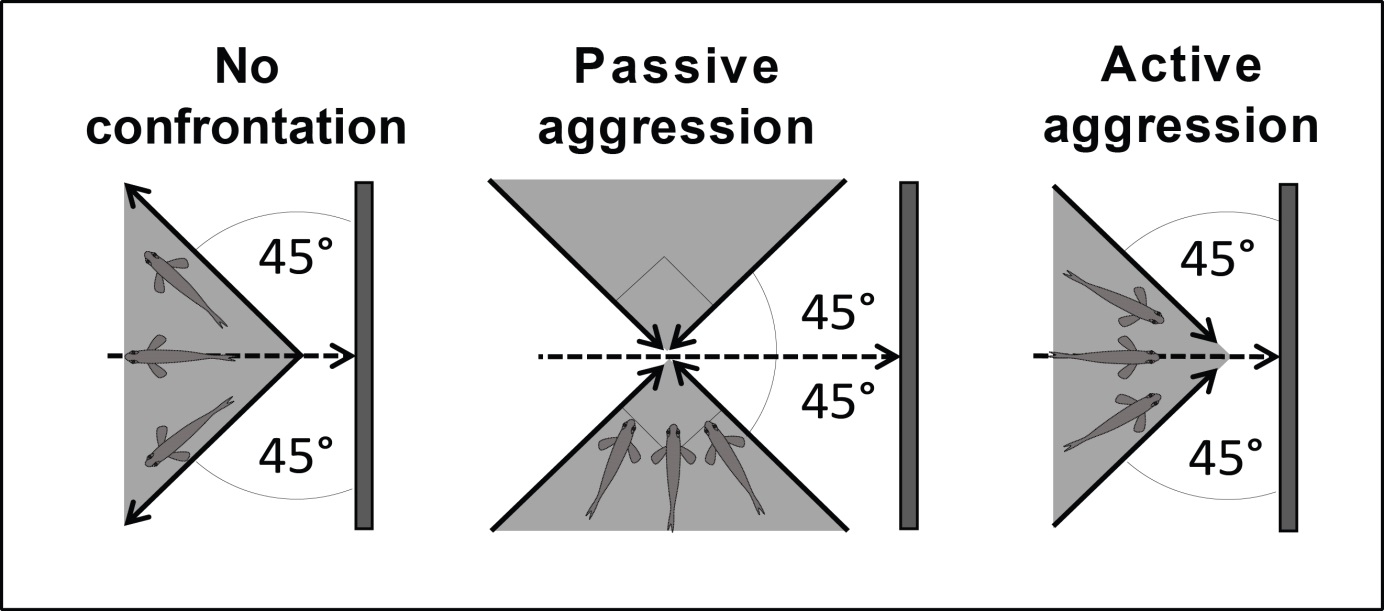
**

**Figure S2.** Definitions of aggression based on fish position relative to the mirror within the confrontation zone. If the fish was inside this zone with its head, then it was scored as confronting the mirror reflection, except for when the body was facing away from the mirror at an angle of >45°. If the fish was swimming actively against the mirror, or swimming towards the mirror at an angle of >45° inside the zone, then the behavior was scored as being actively aggressive (“*AAggr*”). If the fish was inside the zone but not moving, or being faced toward the mirror at an angle ≤45°, or ≤45° away from the mirror, then the behavior was scored as passive confrontation (“*PAggr*”)

# Neophobia test: validation

The neophobia test requires the fish to show reactions toward the object presented into the test arena. This may or may not be true.

To test whether the fish in our experiment reacted toward the novel objects used in this study with altered space utilization, we analyzed space usage for each fish using χ^2^-tests. Higher than predicted utilization of Zone 1 (see Fig 2B in main article) would be indicative of neophilia, while higher than predicted utilization of Zone 4, alternatively lower than predicted utilization of Zone 2, would indicate neophobia.

**Trial 1:** 7 out of 90 individuals showed significant (*P* < 0.05) deviations from the expected zone utilization as predicted from random swimming pattern (5 fish classified as neophilic and 2 fish classified as neophobic). See Table S1A.

**Trial 2:** 5 out of 90 individuals showed significant deviations (*P* < 0.05) from the expected zone utilization as predicted from random swimming pattern (1 fish classified as neophilic, 4 fish classified as neophobic). See Table S1B.

In conclusion, relatively few fish showed any deviations from what would be expected from random swimming in the novel-object tests. No single fish was consistently classified as clearly neophobic or neophilic in both trials. Thus, we cannot assume that the fish reacted to the novel objects used in this study. The test is presented in the main article because it was part of the sequence of behavioural tests, and therefore should not be omitted from the methods.

Table S1. Summary of the individuals classified as clearly neophobic or neophilic in either trial 1 (A) or trial 2 (B). A fish was regarded as clearly neophobic or neophilic if there was a significant deviation from expected zone utilization, as evaluated by a χ^2^ –test (critical χ^2^ – value, P < 0.05: 43).

|  | | **Zone 1** | | **Zone 2+3** | | **Zone 4** |  |  |  |
| --- | --- | --- | --- | --- | --- | --- | --- | --- | --- |
| **Area %** | | 14.8 | | 35.8+37.4 | | 12.0 |  |  |  |
| **Expected counts :** | | 4 | | 22 | | 4 |  |  |  |
|  | |  | |  | |  |  |  |  |
| **A: Trial 1: Observed counts** | | | | | | | | | |
| **ID** | **Zone 1** | | **Zone 2+3** | | **Zone 4** | |  | **χ^2^** | **Classification** |
| **8** | 22 | | 8 | | 0 | |  | 94 | Neophile |
| **12** | 9 | | 17 | | 4 | |  | 7 |  |
| **31** | 21 | | 9 | | 0 | |  | 84 | Neophobe |
| **41** | 0 | | 10 | | 20 | |  | 75 | Neophobe |
| **51** | 8 | | 14 | | 8 | |  | 11 |  |
| **65** | 17 | | 10 | | 3 | |  | 49 | Neophile |
| **85** | 13 | | 9 | | 8 | |  | 32 |  |
| **89** | 25 | | 5 | | 0 | |  | 127 | Neophile |
| **104** | 0 | | 14 | | 16 | |  | 43 | Neophobe |
| **108** | 15 | | 10 | | 5 | |  | 37 |  |
| **117** | 22 | | 8 | | 0 | |  | 94 | Neophile |
|  |  | |  | |  | |  |  |  |
| **B: Trial 2 : Observed counts** | | | | | | | | | |
| **ID** | **Zone 1** | | **Zone 2+3** | | **Zone 4** | |  | **χ^2^** | **Classification** |
| **8** | 2 | | 10 | | 18 | |  | 57 | Neophobe |
| **12** | 0 | | 13 | | 17 | |  | 50 | Neophobe |
| **31** | 5 | | 18 | | 7 | |  | 3 |  |
| **41** | 0 | | 15 | | 15 | |  | 36 |  |
| **51** | 16 | | 10 | | 4 | |  | 43 | Neophile |
| **65** | 4 | | 19 | | 7 | |  | 3 |  |
| **85** | 4 | | 8 | | 18 | |  | 58 | Neophobe |
| **89** | 7 | | 17 | | 6 | |  | 4 |  |
| **104** | 3 | | 27 | | 0 | |  | 5 |  |
| **108** | 4 | | 10 | | 16 | |  | 43 | Neophobe |
| **117** | 6 | | 10 | | 14 | |  | 33 |  |

# Size and growth: detailed statistical results

## Size: wet mass (g) (GLMM)

The general effect of Treatment can be noted (significant contrasts: HL > HH, HL > LH, HL > LL, all *P* < 0.015), but is not particularly interesting in this experimental setting, as it depends largely on initial differences and subsequent assignment of food rations (see Fig. 1a in the main article). The general increase in mass over time (significant contrasts: Date 1 < Date 3, *P* < 0.001; Date 2 < Date 3, *P* < 0.001) is expected. The interaction term Treatment × Date was not significant at the 5% level, but showed a trend (P < 0.1; Table S2). We use non-corrected pairwise contrast estimates to assess differences among groups at the different dates (Table S3). At the start of the experiment (Date 1) HH had, by chance, significantly lower mass than HL. At the switch of rations (Date 2) HH, LH, and LL had lower mass than HL. At the end of the experiment no treatments differed significantly. The groups becoming more similar (contrary to what would be expected) depended on initial differences. Significant differences are marked in bold in Table S3.

**Table S2.** Statistics for main effects and interactions in the generalized linear mixed model (probability distribution: Gaussian; link function: identity) of wet mass over the experiment.

| **Factor** | ***F*** | **df 1** | **df 2** | ***P*** |
| --- | --- | --- | --- | --- |
| Treatment | 4.648 | 3 | 255 | 0.002 |
| Date | 14.331 | 2 | 255 | <0.001 |
| Treatment × Date | 1.942 | 6 | 255 | 0.075 |

**Table S3.** Pairwise contrast comparisons. *P*-values are not corrected for multiple comparisons (least significant difference significance level: 0.05)

| **A: Date 1: Day 0 – Start of feeding treatments**   \|  \| **HH** \| **HL** \| **LH** \| **LL** \|  \| \| --- \| --- \| --- \| --- \| --- \| --- \| \| **HH** \|  \| -0.05 \| -0.01 \| -0.05 \| **Contrast estimates** \| \| **HL** \| **0.04** \|  \| 0.04 \| 0.01 \| \| **LH** \| 0.64 \| 0.13 \|  \| -0.04 \| \| **LL** \| 0.07 \| 0.86 \| 0.19 \|  \| \|  \| ***P* - value** \| \| \| \|  \| | **B: Date 2: Day 12 – Switch of rations**   \|  \| **HH** \| **HL** \| **LH** \| **LL** \|  \| \| --- \| --- \| --- \| --- \| --- \| --- \| \| **HH** \|  \| -0.05 \| 0.04 \| 0.01 \| **Contrast estimates** \| \| **HL** \| **0.03** \|  \| 0.09 \| 0.07 \| \| **LH** \| 0.06 \| **<0.01** \|  \| -0.03 \| \| **LL** \| 0.54 \| **<0.01** \| 0.23 \|  \| \|  \| ***P* – value** \| \| \| \|  \| \|  \|  \| \| \| \|  \| |
| --- | --- | --- | --- | --- | --- | --- | --- | --- | --- | --- | --- | --- | --- | --- | --- | --- | --- | --- | --- | --- | --- | --- | --- | --- | --- | --- | --- | --- | --- | --- | --- | --- | --- | --- | --- | --- | --- | --- | --- | --- | --- | --- | --- | --- | --- | --- | --- | --- | --- | --- | --- | --- | --- | --- | --- | --- | --- | --- | --- | --- | --- | --- | --- | --- | --- | --- | --- | --- | --- | --- | --- | --- | --- |
| **C: Date 3: Day 34 – End of feeding treatments**   \|  \| **HH** \| **HL** \| **LH** \| **LL** \|  \| \| --- \| --- \| --- \| --- \| --- \| --- \| \| **HH** \|  \| 0.00 \| 0.02 \| 0.04 \| **Contrast estimates** \| \| **HL** \| 0.97 \|  \| 0.02 \| 0.04 \| \| **LH** \| 0.38 \| 0.36 \|  \| 0.02 \| \| **LL** \| 0.09 \| 0.08 \| 0.40 \|  \| \|  \| ***P* - value** \| \| \| \|  \| |  |

## Growth rate: *SGR_M_*, specific growth rate (wet mass, % per day) (GLMs)

Growth rates over Period 1 and Period 2 were analysed separately to include initial size (fork length) at the start of each period in the models. Both models were initially run with the interaction Treatment × Initial size. These interaction terms were subsequently removed as they were non-significant (Period 1: P =0.476; Period 2: P = 0.847). Treatment had significant effects on growth rate in both periods (Table S4), following the expected patterns (see Fig. 1B in the main article). Body size (fork length) had significant effects on growth rate, with smaller fish growing faster than relatively larger fish.

**Table S4.** Statistics for the generalized linear models (probability distribution: Gaussian; link function: identity) of specific growth rate over Period 1 (A, B) and Period 2 (C, D). P-values from pairwise comparisons (B, D) are not corrected for multiple comparisons (least significant difference significance level: 0.05). *** = < 0.001.

| **A: Period 1 – Main effect statistics**   \| **Factor** \| $\boldsymbol{\chi}_{\boldsymbol{Wald}}^{\boldsymbol{2}}$ \| **df** \| ***P*** \| \| --- \| --- \| --- \| --- \| \| Treatment \| 145.198 \| 3 \| < 0.001 \| \| Initial size  (9 Jun)* \| 56.749 \| 1 \| < 0.001 \|   * Parameter estimate:  B = -0.0019 (95% CI: ±0.00048) | **B: Pairwise comparisons of estimated marginal means – Period 1**   \|  \| \| **HH** \| \| **HL** \| **LH** \| **LL** \|  \| \| \| --- \| --- \| --- \| --- \| --- \| --- \| --- \| --- \| --- \| \| **HH** \| \|  \| \| -0.001 \| 0.018 \| 0.017 \| **Mean difference** \| \| \| **HL** \| \| 0.76 \| \|  \| 0.018 \| 0.018 \| \| **LH** \| \| ******* \| \| ******* \|  \| 0.000 \| \| **LL** \| \| ******* \| \| ******* \| 0.92 \|  \| \|  \|  \| \| ***P* – value** \| \| \| \| \|  \| \| |
| --- | --- | --- | --- | --- | --- | --- | --- | --- | --- | --- | --- | --- | --- | --- | --- | --- | --- | --- | --- | --- | --- | --- | --- | --- | --- | --- | --- | --- | --- | --- | --- | --- | --- | --- | --- | --- | --- | --- | --- | --- | --- | --- | --- | --- | --- | --- | --- | --- | --- | --- | --- | --- | --- | --- | --- | --- | --- | --- | --- | --- | --- | --- |
| **C: Period 2 – Main effect statistics**   \| **Factor** \| $\boldsymbol{\chi}_{\boldsymbol{Wald}}^{\boldsymbol{2}}$ \| **df** \| ***P*** \| \| --- \| --- \| --- \| --- \| \| Treatment \| 112.238 \| 3 \| < 0.001 \| \| Initial size  (21 Jun)* \| 38.597 \| 1 \| < 0.001 \|   * Parameter estimate:  B = -0.0008 (95% CI: ±0.00024) | **D: Pairwise comparisons of estimated marginal means – Period 2**   \|  \| **HH** \| **HL** \| **LH** \| **LL** \|  \| \| --- \| --- \| --- \| --- \| --- \| --- \| \| **HH** \|  \| 0.006 \| -0.004 \| 0.003 \| **Mean difference** \| \| **HL** \| ******* \|  \| -0.010 \| -0.003 \| \| **LH** \| ******* \| ******* \|  \| 0.007 \| \| **LL** \| **0.006** \| **0.001** \| ******* \|  \| \|  \| ***P* - value** \| \| \| \|  \| |

## Total growth: absolute growth in wet mass (g) (GLM)

Absolute growth in wet mass (g) over the whole experiment was analyzed using initial size (fork length) as a covariate. The model was initially run with the interaction Treatment × Initial size. This interaction term was significant and retained in the model (Table S5). Treatment had significant effects on growth (Table S5; see also Fig. 1C in the main article). Initial body size (fork length) had a significant effect on growth, with smaller fish growing more than relatively larger fish.

**Table S5.** Statistics for the generalized linear models (A) (probability distribution: Gaussian; link function: identity) of total growth in body wet mass (g). P-values from pairwise comparisons (B) are not corrected for multiple comparisons (least significant difference significance level: 0.05). *** = < 0.001.

| **A: Main effect statistics**   \| **Factor** \| $\boldsymbol{\chi}_{\boldsymbol{Wald}}^{\boldsymbol{2}}$ \| **d.f** \| ***P*** \| \| --- \| --- \| --- \| --- \| \| Treatment \| 5.389 \| 3 \| 0.145 \| \| Initial size* \| 28.139 \| 1 \| < 0.001 \| \| Treatment × Initial size † \| 8.858 \| 3 \| 0.031 \|   * Parameter estimate:  B = -0.0088 (95% CI: ±0.0046)  † Parameter estimates:  HH × Initial size: B = 0.0070 (95% CI: ±0.0062)  HL × Initial size: B = -0.0005 (95% CI: ±0.0065)  LH × Initial size: B = 0.0053 (95% CI: ±0.0061)  LL × Initial size: B = 0 (Redundant) | **B: Pairwise comparisons of estimated marginal means**   \|  \| **HH** \| **HL** \| **LH** \| **LL** \|  \| \| --- \| --- \| --- \| --- \| --- \| --- \| \| **HH** \|  \| 0.045 \| 0.033 \| 0.081 \| **Mean difference** \| \| **HL** \| ******* \|  \| -0.011 \| 0.037 \| \| **LH** \| ******* \| 0.24 \|  \| 0.048 \| \| **LL** \| ******* \| ******* \| ******* \|  \| \|  \| ***P* – value** \| \| \| \|  \| |
| --- | --- | --- | --- | --- | --- | --- | --- | --- | --- | --- | --- | --- | --- | --- | --- | --- | --- | --- | --- | --- | --- | --- | --- | --- | --- | --- | --- | --- | --- | --- | --- | --- | --- | --- | --- | --- | --- | --- | --- | --- | --- | --- | --- | --- | --- | --- | --- | --- | --- | --- |

## Size: fork length (mm) (GLMM)

The general effect of Treatment can be noted (HL > LH, HL > HH, both *P* < 0.03), but is not particularly interesting in this experimental setting, as it depends largely on initial differences and subsequent assignment of food rations (see Fig. 1d in the main article). The general increase in mass over time (significant contrasts: Date 1 < Date 3, *P* < 0.001; Date 2 < Date 3, *P* < 0.001) is expected. The interaction term Treatment × Date was not significant (Table S6). Nevertheless, we use non-corrected pairwise contrast estimates of Treatment × Date to assess differences among groups at the different dates (Table S7). The only difference in length detected in the pairwise contrasts was between HL > LH at Date 2. Significant differences are marked in bold in Table S7.

**Table S6.** Statistics for main effects and interactions in the generalized linear mixed model (probability distribution: Gaussian; link function: identity) of fork length over the experiment.

| **Factor** | ***F*** | **df 1** | **df 2** | ***P*** |
| --- | --- | --- | --- | --- |
| Treatment | 3.073 | 3 | 255 | 0.028 |
| Date | 16.836 | 2 | 255 | <0.001 |
| Treatment × Date | 1.178 | 6 | 255 | 0.319 |

**Table S7.** Pairwise contrast comparisons. *P*-values are not corrected for multiple comparisons (least significant difference significance level: 0.05)

| **A: Date 1: Day 0 – Start of feeding treatments**   \|  \| **HH** \| **HL** \| **LH** \| **LL** \|  \| \| --- \| --- \| --- \| --- \| --- \| --- \| \| **HH** \|  \| -1.6 \| -0.3 \| -1.6 \| **Contrast estimates** \| \| **HL** \| 0.09 \|  \| 1.2 \| 0.0 \| \| **LH** \| 0.72 \| 0.18 \|  \| -1.3 \| \| **LL** \| 0.09 \| 0.98 \| 0.18 \|  \| \|  \| ***P* - value** \| \| \| \|  \| \|  \|  \| \| \| \|  \| | **B: Date 2: Day 12 – Switch of rations**   \|  \| **HH** \| **HL** \| **LH** \| **LL** \|  \| \| --- \| --- \| --- \| --- \| --- \| --- \| \| **HH** \|  \| -1.6 \| 0.7 \| -0.5 \| **Contrast estimates** \| \| **HL** \| 0.06 \|  \| 2.3 \| 1.1 \| \| **LH** \| 0.44 \| **0.01** \|  \| -1.1 \| \| **LL** \| 0.58 \| 0.19 \| 0.19 \|  \| \|  \| ***P* – value** \| \| \| \|  \| |
| --- | --- | --- | --- | --- | --- | --- | --- | --- | --- | --- | --- | --- | --- | --- | --- | --- | --- | --- | --- | --- | --- | --- | --- | --- | --- | --- | --- | --- | --- | --- | --- | --- | --- | --- | --- | --- | --- | --- | --- | --- | --- | --- | --- | --- | --- | --- | --- | --- | --- | --- | --- | --- | --- | --- | --- | --- | --- | --- | --- | --- | --- | --- | --- | --- | --- | --- | --- | --- | --- | --- | --- | --- | --- |
| **C: Date 3: Day 34 – End of feeding treatments**   \|  \| **HH** \| **HL** \| **LH** \| **LL** \|  \| \| --- \| --- \| --- \| --- \| --- \| --- \| \| **HH** \|  \| 0.0 \| 0.78 \| 1.1 \| **Contrast estimates** \| \| **HL** \| 0.99 \|  \| 0.74 \| 1.1 \| \| **LH** \| 0.34 \| 0.33 \|  \| 0.6 \| \| **LL** \| 0.15 \| 0.15 \| 0.47 \|  \| \|  \| ***P* - value** \| \| \| \|  \| |  |

## Growth rate: *ΔL,* absolute growth rate in fork length (mm per day) (GLMs)

Absolute growth rates in fork length (mm per day) over Period 1 and Period 2 were analyzed separately to include initial size (fork length) at the start of each period in the models. Both models were initially run with the interaction Treatment × Initial size. These interaction terms were subsequently removed as they were non-significant (Period 1: P =0.201; Period 2: P = 0.926). Treatment had significant effects on growth rate in both periods (Table S8), following the expected patterns (see Fig. 1E in the main article). Body size (fork length) had significant effects on growth rate, with smaller fish growing faster than relatively larger fish.

**Table S8.** Statistics for the generalized linear models (probability distribution: Gaussian; link function: identity) of absolute growth rate (fork length, mm per day) over Period 1 (A, B) and Period 2 (C, D). P-values from pairwise comparisons (B, D) are not corrected for multiple comparisons (least significant difference significance level: 0.05). *** = < 0.001.

| **A: Period 1 – Main effect statistics**   \| **Factor** \| $\boldsymbol{\chi}_{\boldsymbol{Wald}}^{\boldsymbol{2}}$ \| **df** \| ***P*** \| \| --- \| --- \| --- \| --- \| \| Treatment \| 158.668 \| 3 \| < 0.001 \| \| Initial size  (9 Jun)* \| 33.17 \| 1 \| < 0.001 \|   * Parameter estimate: B = -0.0067 (95% CI: ±0.0023) | **B: Pairwise comparisons of estimated marginal means – Period 1**   \|  \| **HH** \| **HL** \| **LH** \| **LL** \|  \| \| --- \| --- \| --- \| --- \| --- \| --- \| \| **HH** \|  \| -0.014 \| 0.081 \| 0.082 \| **Mean difference** \| \| **HL** \| 0.17 \|  \| 0.094 \| 0.096 \| \| **LH** \| ******* \| ******* \|  \| 0.002 \| \| **LL** \| ******* \| ******* \| 0.86 \|  \| \|  \| ***P* – value** \| \| \| \|  \| |
| --- | --- | --- | --- | --- | --- | --- | --- | --- | --- | --- | --- | --- | --- | --- | --- | --- | --- | --- | --- | --- | --- | --- | --- | --- | --- | --- | --- | --- | --- | --- | --- | --- | --- | --- | --- | --- | --- | --- | --- | --- | --- | --- | --- | --- | --- | --- |
| **C: Period 2 – Main effect statistics**   \| **Factor** \| $\boldsymbol{\chi}_{\boldsymbol{Wald}}^{\boldsymbol{2}}$ \| **df** \| ***P*** \| \| --- \| --- \| --- \| --- \| \| Treatment \| 84.381 \| 3 \| < 0.001 \| \| Initial size  (21 Jun)* \| 34.596 \| 1 \| < 0.001 \|   * Parameter estimate: B = -0.0069 (95% CI: ±0.0023) | **D: Pairwise comparisons of estimated marginal means – Period 2**   \|  \| **HH** \| **HL** \| **LH** \| **LL** \|  \| \| --- \| --- \| --- \| --- \| --- \| --- \| \| **HH** \|  \| 0.062 \| 0.008 \| 0.068 \| **Mean difference** \| \| **HL** \| ******* \|  \| -0.054 \| 0.007 \| \| **LH** \| 0.42 \| ******* \|  \| 0.061 \| \| **LL** \| ******* \| 0.48 \| ******* \|  \| \|  \| ***P* - value** \| \| \| \|  \| |

## Total growth: absolute growth in fork length (mm) (GLM)

Absolute growth in fork length over the whole experiment was analyzed using initial size (fork length) as a covariate. The model was initially run with the interaction Treatment × Initial size. This interaction term was subsequently removed as it was non-significant (*P* =0.886). Treatment had significant effects on growth (Table S9; see also Fig. 1F in the main article). Initial body size (fork length) had significant effects on growth rate, with smaller fish growing more than relatively larger fish.

**Table S9**. Statistics for the generalized linear models (A) (probability distribution: Gaussian; link function: identity) of total growth in fork length (mm). P-values from pairwise comparisons (B) are not corrected for multiple comparisons (least significant difference significance level: 0.05). *** = < 0.001.

| **A: Main effect statistics**   \| **Factor** \| $\boldsymbol{\chi}_{\boldsymbol{Wald}}^{\boldsymbol{2}}$ \| **df** \| ***P*** \| \| --- \| --- \| --- \| --- \| \| Treatment \| 83.521 \| 3 \| < 0.001 \| \| Initial size* \| 60.740 \| 1 \| < 0.001 \|   * Parameter estimate:  B = -0.23 (95% CI: ±0.057) | **B: Pairwise comparisons of estimated marginal means**   \|  \|  \| ***J*** \| \| \| \|  \| \| --- \| --- \| --- \| --- \| --- \| --- \| --- \| \|  \|  \| **HH** \| **HL** \| **LH** \| **LL** \|  \| \|  \| **HH** \|  \| 1.20 \| 0.98 \| 2.33 \| **Mean difference**  **(*I* – *J*)** \| \| ***I*** \| **HL** \| ******* \|  \| -0.22 \| 1.13 \| \|  \| **LH** \| ******* \| 0.38 \|  \| 1.34 \| \|  \| **LL** \| ******* \| ******* \| ******* \|  \| \|  \|  \| ***P* – value** \| \| \| \|  \| |
| --- | --- | --- | --- | --- | --- | --- | --- | --- | --- | --- | --- | --- | --- | --- | --- | --- | --- | --- | --- | --- | --- | --- | --- | --- | --- | --- | --- | --- | --- | --- | --- | --- | --- | --- | --- | --- | --- | --- | --- | --- | --- | --- | --- | --- | --- | --- | --- | --- | --- | --- | --- | --- | --- | --- | --- | --- | --- | --- | --- |

# Complementary analyses of behavior

## Growth rate vs. behavior: analyses using growth rate as continuous factor

**
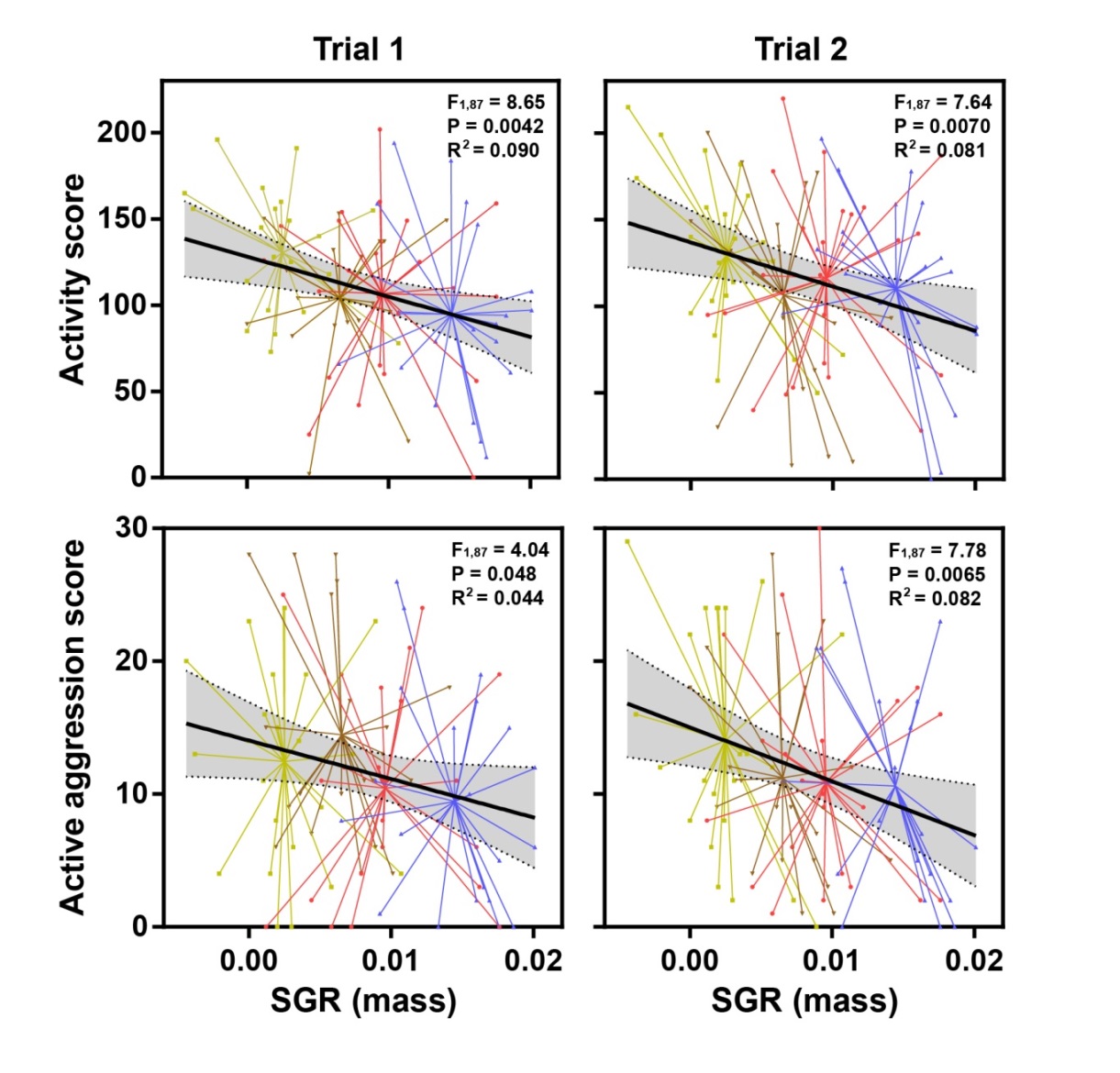
**

**Figure S2.** Regression analyses of behavior using specific growth rate over days 13 - 34 (Period 2) of the experiment as independent variable. For activity and active aggression there were weak, but significant, general negative effects of growth rate Analyses for neophobia scores were non-significant, and are not illustrated here (Trial 1: F_1,87_ = 1.778, P = 0.186, R^2^ = 0.009; Trial 2: F_1,87_ = 0.029, P = 0.866, R^2^ = 0.0003). Treatments are colored for illustrative purposes: green = HL, brown = LL, red = HH, blue = LH. Lines radiate out to data points from the centroid for each treatment. Grey-shaded areas show the 95% confidence intervals for the regression lines.

## Mirror confrontation scores: total confrontation, passive- and active aggression


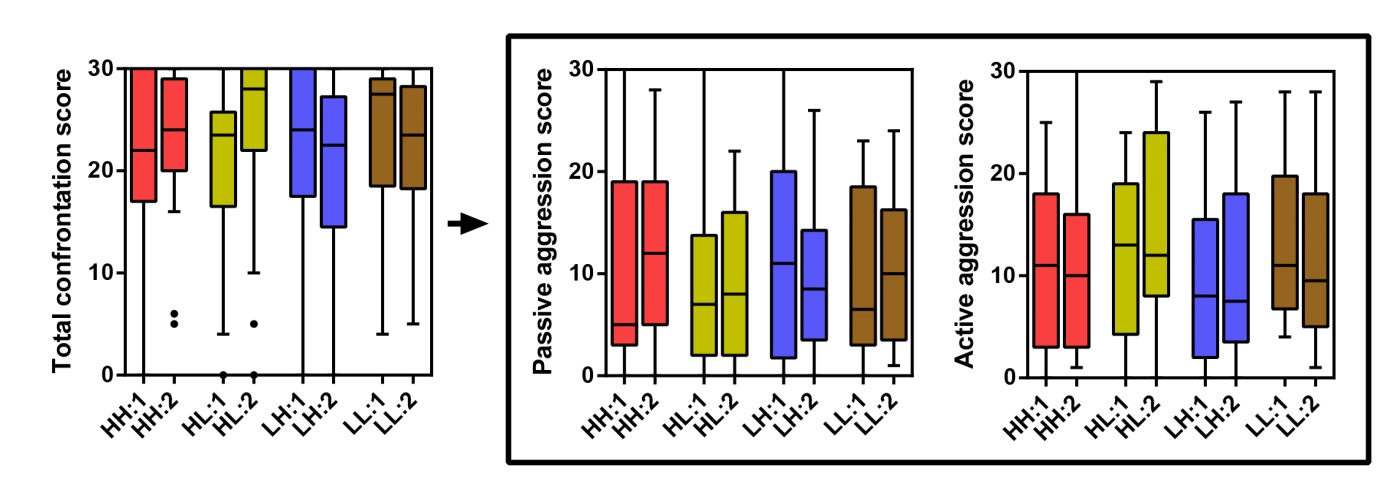


**Figure S3.** Aggression levels for each treatment, each trial (denoted 1 and 2). Total confrontation score is the addition of passive and active aggression scores. Data is shown as Tukey boxplots, with box hinges representing first and third quartiles and the band within the box represents the second quartile (median). Whiskers represent the data within 1.5 interquartile range from the box hinges and dots represent data points more than 1.5 interquartile range away from the box hinges. Note the high level of total aggression in the total confrontation score; the majority of all fish are located within the confrontation zone, either passively displaying or actively swimming towards the mirror image at > 66% of all scoring moments.

## Cluster assignment in relation to initial and final size


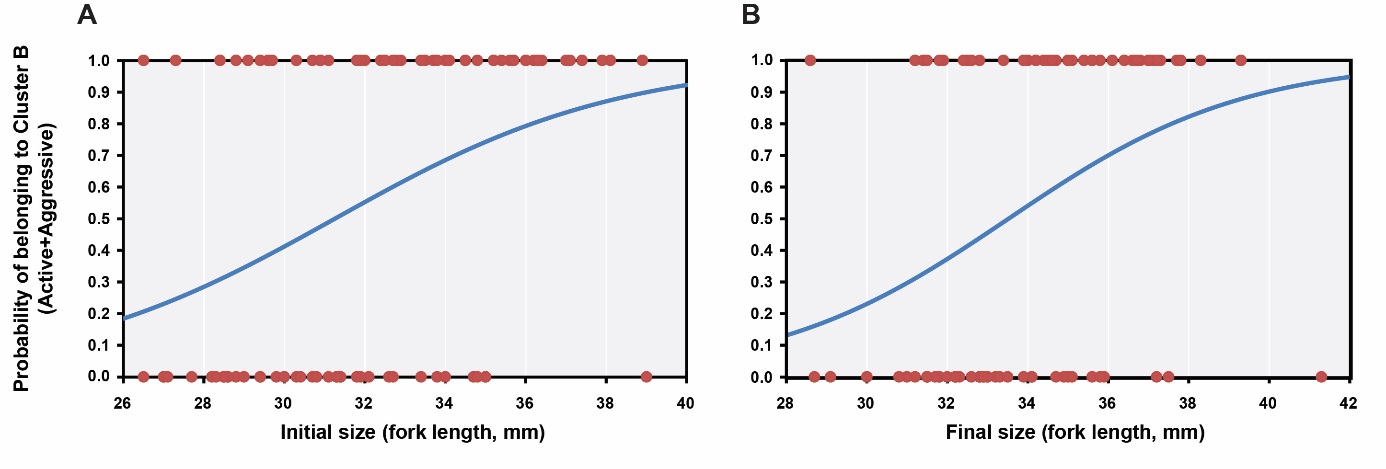


**Figure S4.** Probabilities of being assigned to Cluster B (active and aggressive) as predicted by binary logistic GLM using initial size at the start of the experiment (A) and final size at the end of the feeding manipulations (B) as independent variables. Functions for the regression lines are: (A) $y=1/\left( 1+e^{\left( 8.845-0.283x \right)} \right)$, and (B) $y=1/\left( 1+e^{\left( 11.464-0.342x \right)} \right)$. Omnibus tests were significant for both models (*P* < 0.001).
